# Fear of panoptic surveillance: using digital technology to control the COVID-19 epidemic

**DOI:** 10.1186/s13584-020-00429-7

**Published:** 2020-11-25

**Authors:** Yael Keshet

**Affiliations:** Western Galilee College, Hamichlala Road, 2412101 Akko, Israel

**Keywords:** COVID-19, Contact tracing, Digital technology, Surveillance technologies

## Abstract

**Background:**

In a bid to reduce infection rates by COVID-19 the authorities in some countries, in collaboration with medical regulators and experts, have employed digital technologies to control and regulate citizens’ behavior. Public opinion and the public’s compliance with these technologies come into play here. The objective of the present study was to examine attitudes expressed in the public discourse toward the use of digital technologies to control people’s behavior during the COVID-19 pandemic, as reflected in the media.

**Methods:**

Qualitative analysis was performed on posts and comments submitted in response to 12 articles that appeared on the four leading Israeli news sites, on three significant occasions: first, upon the announcement of the use of surveillance technologies by the Israeli security agency (ISA); second, upon the announcement of the launch of the Health Ministry’s app that tracks contacts with COVID-19 patients; and third, following reports of petitions lodged with Israel’s supreme court challenging the use of surveillance technologies. The analysis was performed using ATLAS-Ti software for systematic analysis.

**Results:**

A total of 2551 posts and comments referring to these 12 articles were found, 714 of which were relevant to the purpose of the study. The analysis revealed disagreement between those who supported the measures taken and opponents. Supporters regarded contact tracing by means of digital technologies as essential to the effort to protect people during the pandemic, and believed that employing the ISA’s capabilities was the correct way to combat the epidemic. Opponents of the measures rejected the use of tracking technologies, regarding this step as a move toward dictatorship and a violation of fundamental civil rights. Some proposed alternative measures that would obviate the use of such tracking.

**Conclusions:**

The primary task of medical practice is to heal illness and alleviate suffering. The debate surrounding the employment of digital technologies during the COVID-19 pandemic highlights the complexity of the close connection between social control and care in times of pandemic. The context of this pandemic has highlighted the interrelatedness of advanced digital surveillance technologies, medical care, and social control exercised by authorities and medical regulators and experts, which raises issues of transparency, trust and mistrust among the public. These issues become all the more relevant when the number of patients grows rapidly, the authorities need to deal with the extended ongoing COVID-19 pandemic, the country has entered a second lockdown, and the public must be persuaded to comply with highly restrictive regulations. Recommendations to policy makers, practical implications, and suggestions for future research are discussed.

## Background

The COVID-19 pandemic is a major event, and diverse steps have been taken to contain and control it. These steps, such as the formulation of hygiene norms, maintaining physical distance, and isolation are intended to curb the spread of the virus and thereby reduce fatalities and morbidity. The need to ensure physical distancing and to interrupt chains of infection so as to reduce contagion led to the imposition of social control effected through collaboration between governments, medical experts, and regulators. Since the pandemic struck in the era of advanced digitization, available digital technologies have allowed the authorities in many countries to enhance surveillance and to control people’s behavior to further medical objectives. These technologies enable the authorities to collect vast amounts of information on individuals’ contacts, which far exceeds that regularly obtained by health authorities [3]. Since the public’s cooperation with these measures is vital, public opinion and the public’s compliance with these technologies come into play here.

### Medical experts and medical regulators

In his famous series of lectures, which became known as *Society Must Be Defended* ([9] (1975–1976)), Foucault distinguished between two types of medical domain. The first treats individuals and their bodies in practical terms, based on medical knowledge; and the second deals with the regulation of social life –controlling the population with the aim of reducing morbidity and mortality. “Medicine is a power-knowledge that can be applied to both the body and the population, both the organism and biological processes, and it will therefore have both disciplinary effects and regulatory effects” ([9]:252).

The two types of medical practice were established and superimposed from the end of the eighteenth century onward: the disciplinary track, based on medical knowledge, research, and the practice of health care, centers on the body and produces individualizing effects. The regulatory process focuses not upon the body but upon the life of a population, and “tries to control the series of random events that can occur in a living mass, a technology which tries to predict the probability of those events (by modifying it, if necessary), or at least to compensate for their effects.” ([9]:249).

The power to regulate the health of a population, which Foucault termed bio-politics, leads authorities to treat the health of a population primarily as a political problem that also has biological and scientific aspects. The mechanisms introduced by bio-politics include forecasts, statistical estimates, and overall measures. They seek to impact outcomes at the population level, such as lowering mortality and morbidity rates. To accomplish this, control and regulatory mechanisms must be established. This is the power of the sovereign, which Foucault [9] regarded as the right to intervene to regulate health. Medical knowledge and practice, on the one hand, and the regulation of populations on the other, these two heterogeneous layers of discipline and sovereignty, converged in the efforts to combat the pandemic.

### Digital contact tracing and privacy

One of the regulatory measures taken to contain and control COVID-19 was to use digital technologies that facilitate the tracing of contacts between diagnosed patients and other individuals during the 14 days prior to the date on which the patient was diagnosed with the disease. By informing every individual who has been in contact with a person infected with COVID-19 and isolating them, the spread of the virus can be slowed down. However, while digital contact tracing using smartphones has been proposed as a scalable and efficient tool to break chains of infections, this method impinges on individuals’ privacy [4]. The obvious risk is related to the potential misuse of the large amount of detailed sensitive information collected pertaining to people’s contacts and connections with others [3]. Tracing through individuals’ smartphones may expose confidential information about their location history, meeting history, and health condition. It is important not to disclose a person’s medical data to others without their prior consent. People may not want to disclose this information to someone they do not trust [3], or may fear being discriminated against or stigmatized because of their health condition [4]. People should have the right to control who, when, and where someone else gains access to their data [3].

Furthermore, if digital contact tracing is to achieve its intended purpose, the system infrastructure must collect vast amounts of information about as many people’s contacts as possible. Tracing so many citizens is considered inappropriate in democratic societies. Therefore, effective contact tracing should be conducted, while minimizing the exposure of people’s sensitive data [3]. “The contact tracing technologies used in a number of Asian countries collect highly sensitive data from individuals. However, data protection and privacy regulations, especially with respect to medical data, may significantly differ from country to country. For example, in several European countries or in the US, there are more restrictive regulations.” ([3]:3). Researchers and governmental agencies in Europe and the USA are currently investing efforts into developing digital technologies that maintain an appropriate level of security, privacy and transparency. For example, both the Swiss and the Germans have developed apps that employ an open source approach, involving the community in early testing of the app code, at least in theory. There has been a lively public debate regarding privacy issues in the EU and USA, while in some other countries they have not been widely discussed [3]. Moreover, while in some countries citizens are asked to install the app voluntarily and must actively use it if it is to be effective, in other countries the tracking technology is mandatory. The way such technologies are installed is closely connected to the prevailing attitude toward the protection of individual privacy [4].

Israel, which has highly developed digital technological capabilities [14] and is known as the “start-up nation” and a leading source of technological innovation worldwide [2], has used both voluntary and mandatory digital technologies to track, control and regulate population behavior during the COVID-19 pandemic.

### The Israeli context

The COVID-19 epidemic broke out in Israel toward the end of February 2020. A lockdown was imposed in mid-March and most restrictions were lifted a month later, in mid-April. Between April and early June, the COVID-19 infection rate in Israel remained stable but then began to rise and in late September the country entered a second nationwide lockdown.

As early as March17, 2020, the government passed a set of emergency regulations authorizing the Israel Security Agency (ISA) to aid in the national effort to limit the spread of the COVID-19, and permitting it to collect, process, and use of citizens’ personal data. The ISA started using a cache of mobile-phone-location data to help identify people who had crossed paths with patients who tested positive for COVID-19. People found to have been in close contact with an infected individual were placed into mandatory quarantine to stop further contagion [1]. Six days after the initiation of mandatory COVID-19 surveillance by the ISA, the Ministry of Health launched a very similar but optional service named HaMagen (the protector), an open-code application that allows citizens to opt in to the logging of their mobile-phone locations. This application notifies mobile users shortly after they come into contact with a person who has tested positive for COVID-19 (as recorded by health officials) and advises them to self-isolate [1]. A public debate ensued in Israel regarding the use of these surveillance technologies.

### The research objective

The study sought to examine attitudes expressed in the public discourse (as reflected in the media) toward the use of digital surveillance technologies to monitor population behavior during the COVID-19 pandemic.

## Methods

Qualitative analysis was performed on posts and comments submitted in response to 12 articles that appeared on three significant dates on the four leading Israeli news sites. The Posts and Comments section is a feature of news websites in which readers are invited to comment on the published content. This feature enables readers to share their reactions to the issues reported on news websites. A post generally elicits comments and this initiates a discussion.

Three dates were chosen on which a significant policy announcement or broadcast was made: first, regarding the use of surveillance technologies by the Israeli security agency (ISA), on March17, 2020; second, regarding the launch of the Health Ministry app that tracks contacts with COVID-19 patients, on March22, 2020; and third, a live TV broadcast from Israel’s supreme court as it considered petitions challenging the use of surveillance technologies, on April16, 2020. The data was collected in late April 2020.

The four leading Israeli news sites were sampled according to the SimilarWeb site, which tracks companies’ digital market share. The four top news and media websites in Israel are: ynet.co.il (21.3% market share in the category), walla.co.il (11.4%), maco.co.il (10.1%), and Haaretz.co.il (3.4%). Since the four leading Israeli news sites publish in Hebrew, almost all of the posts and comments were also written in Hebrew. Thus, our analysis is relevant only to the Hebrew speaking population. The total of 2551 posts and comments referred to the 12 news articles, of which 28% were directly relevant to the research objective. The remainder addressed the political situation that pertained at that time in Israel or were non-sense posts.

Thus, the data that was analyzed comprised posts and comments written at different times, submitted to different news websites by many different individuals. This combination produces “data triangulation,” one of the four types of triangulation that Denzin [6] identified. Data triangulation has three subtypes: (a) time, (b) space, and (c) persons. Accordingly, data triangulation refers to data collected at different points in time, from different people, and at different intervals, so as to obtain a richer and more detailed picture of the phenomena. Variance in events, settings, times, and so forth may bring to light revealing atypical data or recurrent patterns, both of which enhance confidence in the findings [5].

Content analysis of the relevant posts and comments was conducted according to the procedure proposed by Keshet et al. [15] for short narratives analysis. This is a qualitative mode of content analysis, since it focuses on meanings rather than measurements [12]. Content analysis is defined as a research technique applied to non-statistical material that allows the researcher to analyze such material in a systematic manner [8, 20].

The posts and comments were coded systematically using Atlas.ti Scientific Software version 8. The ATLAS.ti software enables one to develop a coding schema that indicates the topics or concepts that emerge from the data. This involves selecting quotations and assigning them a code, after which all quotations with the same code are retrieved by running a report produced by the code manager. This process facilitates retrieval of related quotations in order to examine patterns in the data, and enables the grouping of codes into categories that represent broader and more abstract themes.

Analyzing posts and comments on website news articles offers several advantages but also entails disadvantages in comparison to other qualitative methods such as in-depth interviews and focus groups. Post and comments analysis better reflects opinions in the public discourse since they capture the authentic responses of people to news in the media because they respond immediately and more freely by virtue of the anonymity that this medium allows. Furthermore, this method provides access to a very large volume of opinions and attitudes expressed at the same time, which is obviously not the case when in-depth interviews are conducted. On the other hand, this method tells us nothing about the person who posted or commented and we cannot ask follow-up questions to gain further insights into each individual.

## Results

The analysis produced seven main codes that were grouped into two broader and more abstract themes: supporters and opponents (Table 1). Supporters consider these measures to be vital to dealing effectively with the epidemic, as they save people’s lives. They believe that employing the ISA’s capabilities is the correct way to fight the pandemic. Concerns were expressed about those who do not own smart phones. However, many posts and comments expressed opposition to the use of digital surveillance technologies, as this move was considered a dangerous step toward dictatorship and a violation of fundamental rights. They furthermore noted ways whereby individuals could avoid surveillance.

**Table 1:** Theme and codes

Themes	Codes
Supporters	Tracking is important
	Nothing to hide
	Do not own a smart phone
Opponents	Danger of dictatorship
	Violation of the right to privacy
	Ways to evade tracking
	Anyhow no privacy in the digital environment

### Supporters: “Health and public safety are more important than privacy”

Posts and comments that support the use of surveillance technology emphasize its positive intent. “The move is designed to locate people who have been in close contact with a sick person before knowing about it, and thus reduce the spread of the disease. It seems an important step.”

When the Israeli Ministry of Health endorsed the HaMagen app someone posted “I’ve downloaded it myself, and I urge you to download.” And someone else explained: “To fight the virus and because the information patients report cannot be relied upon; I also can’t remember exactly where I was and at what time, [so] I installed the protective app and gave permission to follow me and it helps me, if I’m near a sick person, I’d rather lose privacy.” Another posted that “we have to follow those who need isolation, because those who violate it pose a danger! They are irresponsible.”

When the supreme court addressed the petitions lodged against the use of ISA surveillance technologies, some wrote that “public health and safety are more important than privacy”; that “the government is working to save all its citizens, in the wake of a global deadly epidemic... No more self-destruction,” adding that ISA data collection “is only for the purpose of the Corona and for no other use.” Some wrote that since we were now at war (against the COVID-19) ISF intervention was justified:

and “they shall beat their swords into plowshares, and their spears into pruning hooks,” taking tools of war and turning them into a tool of peace. This is a technological tool that can stop the epidemic immediately. Very soon, we will emerge from the crisis thanks to this directive.

Others argued that using this tool did no damage to democracy: “Democracy can bear such harsh tools, one shouldn’t be afraid of it, one must make sure it is done transparently and with limitations. And at least for now, that seems to be what the state is doing. Judge Meltzer suggested adding more external oversight.” Another comment read: “… I know what the big brother’s eye means but today I have no problem, knowing where I would be if I were near a Corona patient. This is an emergency and these regulations will not be [here] forever.”

Those who responded to opponents’ posts argued that there was nothing to hide. “Why are they afraid? Only those who have something to hide are afraid,” and “… only those who fear the ISF know why they are afraid.” “What has Corona to do with the Supreme Court? Everything must be done by all means to save [life] both forcefully and powerfully.”

Some voiced criticism of the measure, pointing out that surveillance technology applies only to smart phone owners, and that many in certain sections of the population do not own such a devise. This is true mainly of the poor and of ultra-orthodox Jews, who shun smart phones because of their beliefs: “The most problematic population is the ultra-Orthodox population that does not use a cell phone and cannot be monitored...” Another asked “The app does not work on devices below a certain level that most people maintain, you have no shame the Ministry of Health not to think of the poorest among the most vulnerable, we also pay taxes.”

### Opponents: “In the sequel, a chip will be implanted under one’s skin, there will be 24/7 monitoring of every detail”

Opponents of the use of surveillance technologies addressed mainly three issues: the danger of dictatorship, violation of the right to privacy, and ways to avoid surveillance. Commentators perceived the use of surveillance technologies to be “anti-democratic steps [taken] by the government,” and part of the process of eroding democracy: “Democracy in the State of Israel is dying,” “No democratic country has been monitoring cell phones.” While some view this measure as a danger to democracy, others go further to consider it “the end of democracy.” One described Israel “as a totalitarian state” and another as a “police state.” “This attempt to cling to every draconian way of dealing with the epidemic, evokes even more distrust!!! Start behaving like democracies that have chosen more humane ways and achieved better results than Israel.”

Many of those who submitted posts and comments used the term “dictatorship” to clarify their feelings about the move. Some referred to it as “a dictatorship beneath a mask of democracy,” and as “a dark government, a regime of oppression.” The concern raised by several commentators was that “the regime does not abandon tools that allow control after it gets used to them.” They therefore predicted that surveillance measures would be kept in place even once the need evaporated: “Do not be surprised when the ISA monitors you at the end of the COVID-19 crisis, and then surveillance will also be used by the police, and then the surveillance will allow tracing of extreme political activists.”

The ISA’s intention to apply surveillance measures aroused antagonism, expressed in posts and comments. One of these expressed concern regarding a security service that routinely tracks civilians:

These are unique means that the ISA uses for surveillance of Palestinians in the West Bank and the Gaza Strip. It is especially naive to believe that a dark organization like the ISF will not exploit the situation to gather intelligence on civilians.

A commentator expressed his/her discomfort at the lack of transparency: “in the fog-covered (important in themselves) actions of the secret services, a dark regime, this [is a] toxic initiative by the state leadership under the guise of security. Civilian surveillance is a key feature of dark regimes.” And another wrote that “What is real is that they want to imprison us in our homes …. Corona is the biggest lie of the 21^st^ century, meanwhile while we are isolating, [they are] covering the country with the G5 military system for civilians.”

These actions on the part of the regime were likened to dark periods in history. In many posts and comments, the comparison with the rise of Nazism in Germany is striking. Making comparisons to the rise of Nazism evokes strong emotion in Israel. “1932 - this is how it always begins.” “And maybe an electronic chip will be embedded in the back of our neck. Today they no longer have to tattoo a number on one’s arm.” And someone wrote that “Anyone who learns from history understands what’s going on here. Anyone who … didn’t believe it could happen, like my dear family members … was murdered in the Holocaust.”

Commentators also compared the use of surveillance technologies to other regimes at different times in history, such as “the establishment of the Stasi,” “the USSR then,” “the Israeli Ceausescu,” “It is exactly in line with Benito Muslino,” “The KGB is already here,” and “we have almost become a Bolshevik regime.”

Alongside anxiety over the fate of democracy and the fear of moving toward a dictatorial regime, many commentators perceived the use of surveillance technology to deal with the COVID-19 epidemic to be a violation of the right to privacy. “This is a blatant intrusion on privacy, on human rights,” a violation of “the most fundamental right to freedom.” Posts mentioned “a state that violates human rights,” and some claimed that “the government has no right to track its citizens.”

The peculiar circumstances of the pandemic raised awareness of the government’s ability to monitor its citizens: “Unbelievable, they really admit they are able to track us all ... I was sure cellular companies were helping in that, but it turns out that the state can monitor and break into all of our devices .... wow.” Concerns were raised about other uses of this technology that could benefit society, but at a heavy price of violating privacy: “The ISA will … expand the circle without our knowledge, to obtain more information. Perhaps it’s good for a war on crime but definitely very bad for privacy.” Some argued that weighing the right to life against the right to privacy was merely an excuse: “Emergency is prevalent in many countries because of the epidemic, but this does not justify human rights violation...”. A further concern was the leakage of information to be collected, something that had recently occurred in Israel during the elections. “What are the chances that the information will really be erased and not leaked, in light of the very bad experience we had here?”

Some drew parallels with “the Big Brother” literature: “It’s really a bad science fiction movie,” “A life without privacy just like George Orwell warned about in his book 1984,” “In the sequel, a chip will be implanted under the skin, there will be 24/7 monitoring of every detail.”

A number of suggestions were made on how to evade surveillance: turn off the device, take out the SIM card, switch to flight mode, “switch to an old device,” “take out the battery or put the phone in a metal bag.” Leaving the phone at home as a way to escape surveillance was mentioned in many posts, and some declared that they had indeed left the phone at home to evade surveillance, “my cell phone has not been out of the home for a month or so!”

A number of people maintained that we are routinely monitored in a digital environment. They mentioned companies like Google, Facebook, Instagram, and Amazon, as well as credit card companies and cameras: “As if we aren’t already under surveillance,” “What’s new?!!? As if they haven’t been following us already? Ever since the cell phones were invented they have been following us.” Some referred to the tendency of many people to reveal details of their private lives in the media: “What does this exhibitionist generation have to fear, after all, you are (twenty-four seven) exposing yourself to networks, come on!”

## Discussion

This study, which focused on a specific point in time of the spread of the COVID-19 pandemic, raises issues about processes that researchers study over an extended period – in this case, the use of digital technologies to monitor public health and mechanisms of social control. During the outbreak of the COVID-19 pandemic, the processes of monitoring and surveillance became all encompassing.

Digital technologies are commonly used to monitor various medical aspects of our bodies and to facilitate medical supervision in an attempt to prevent illness and disease. These surveillance technologies operate on three different levels: on the individual level, on the interpersonal clinical level, and on the national or global population level [16].

On the individual level, people use self-tracking digital technologies to measure and collect data to promote their health. Some of this self-tracking data is gathered and used for personal purposes, but much of it belongs to internet companies, other commercial entities, or government organizations, and this raises the question to whom the data belongs. The danger in self-tracking is that this very detailed knowledge about oneself may be misused by others, including hackers, perhaps in ways that violate personal autonomy and human rights. On the interpersonal clinical level – the medical encounter – doctors practice a form of personalized surveillance over each of their patients, documented in electronic health records. On the national or global population level, health surveillance systems are used to record and monitor cases of illness and medical conditions such as obesity, in order to track epidemiological changes [18].

From a critical point of view, expressed by those who oppose the use of surveillance technologies, the use of such digital technologies to promote health can be seen as a part of the *surveillance society* [19]. The term surveillance society denotes how digital technology is increasingly being used to monitor everyday life and discipline people. The use of surveillance technologies for healthcare purposes can be seen as a means of exercising power over life, and can be conceptualized in Foucault’s words (2003:253): “We are then in a power that has taken control of both the body and life or that has, if you like, taken control of life in general – with the body as one pole and the population as the other.”

The COVID-19 pandemic has further empowered the surveillance society, as the authorities employ digital technologies to control and regulate citizens’ behavior. The debate between supporters and opponents of the use of digital technologies to track contacts with corona patients highlights their aspects of power and social control. Opponents pronounced concerns about the implications for democracy and civil rights. On the other hand, those who support the use of digital surveillance technologies stress that they save lives. Thus, the COVID-19 pandemic has demonstrated the basic ambiguity of healthcare surveillance – the close connection between control and care, which makes surveillance so complex issue [7, 19].

During the COVID-19 pandemic normal life has been subordinated to medical needs and subjected to restrictions. Many areas of life has came under medical domination, influence, and supervision. Digital technologies that facilitate the identification of contacts require the tracking of individuals’ moves and medical conditions. The nature of the opposition expressed to the imposition of the ISA’s tracing technology evokes fear of panoptic surveillance. Some of the opponents go so far as to regard contact tracing apps not as a solution to the COVID-19 problem, but as the problem itself. The way these opponents perceive the power exercised by such surveillance can be explained by referring to the model of the Panopticon [11]. The word panopticon literally means “all-seeing,” and was used by Jeremy Bentham in the late eighteenth and early nineteenth centuries to denote a particular architectural design. Foucault regarded the panopticon as a means of exercising disciplinary power in modern western societies: “It is the fact of being constantly seen, of being always able to be seen, that maintains the disciplined individual in his subjection” ([10]: 187). Disciplinary principles are expressed in the panopticon: it imposes coercion by means of observation rather than physical violence; it employs training and correction to produce docile, useful, bodies; it promotes the separation of individuals by examining them to produce knowledge of “cases.” The traditional panoptic prison where a few guards were placed in the central tower has evolved into a highly efficient management and surveillance system, with an increasingly sophisticated digital technology capacity for monitoring, data storage, networking, and analysis. This is no longer a matter of surveillance but rather an issue of data analysis. In societies of control the surveillance apparatus does not act on bodies or minds but on information about bodies and minds [22]. Gallagher [11] argued that, in modern societies surveillance has become part of everyday life, inculcated and reinforced by social institutions such as prisons, hospitals, and schools.

Digital surveillance technologies employed during the COVID-19 pandemic can be seen as a type of panopticon put in place by governments in collaboration with medical experts and medical regulators for purposes of curbing the spread of the virus, and thereby reducing fatalities and morbidity. The outbreak of a pandemic highlighted the interrelatedness of information technology exploitation and sophisticated digital surveillance technologies; the exercise of medical social control by authorities in collaboration with medical regulators and experts; transparency; trust and mistrust among the population; and the ambiguity of the close link between control and care – all of which are components of this panoptic- surveillance.

While the occurrence of the epidemic produced an extreme form of panoptic-surveillance, this term can contribute to clarifying processes that play out also in other less dramatic times. It can usefully be applied to the vast body of research on medicalization, such as studies on the distinction between medicalization and over-medicalization (e.g., [13]); research on the use of personalized medicine and digital technologies (e.g., [23]); and studies informed by critical social and cultural theory concerning the use of mobile and wearable health technologies, digital devices, and associated apps, websites, and platforms seeking to promote preventive medicine and public health (e.g., [17, 21]). The present study also has practical implications as a preliminary exploratory research project that addresses the willingness of citizens to cooperate with their government’s measures in times of widespread and global crisis such as a pandemic.

The primary task of medical practice is to heal illness and alleviate suffering. The context of this pandemic has highlighted the interrelatedness of advanced digital surveillance technologies, medical care, and social control exercised by authorities and medical regulators and experts, which raises issues of transparency, trust and mistrust among the public. These issues acquire particular importance given the need to deal with the ongoing enduring COVID-19 pandemic and to gain the public’s compliance with highly restrictive regulations.

To safeguard privacy and civil liberties, a high level of supervision and transparency are recommended. As Amit et al. [1] suggested, the program should be limited in time and be continually re-evaluated. An independent committee comprising experts such as attorneys, ethicists, epidemiologists, and digital privacy experts, as well as representatives of the public should be established to monitor the program daily. Access to data should be limited to as few people as possible. The public should be informed which data is collected, how it will be used, stored, and shared, and be assured that the data will remain anonymous. Health experts should encourage voluntary participation, and device users should consent to the use of their data.

The study has several limitations, which present opportunities for future research. First, it was conducted in a single country over a short time period and analyzed posts and comments that referred to only 12 articles carried by four news sites. The findings therefore highlight a selection of Israelis’ attitudes toward surveillance technology during the COVID-19 pandemic, and cannot be assumed to hold true for other countries. Second, we know nothing about the individuals who submitted the posts and comments and we cannot divulge from this study knowledge of the opinions held by diverse groups within the Israeli population, such as secular and religious individuals, Jews and Arabs, etc. Further research, both quantitative and qualitative (such as in-depth interviews or focus groups), is needed to trace the variety of opinions held and the rates within the population of those who hold them and their willingness to cooperate with the authorities, in Israel as well as in other countries.

## Conclusions

The COVID-19 pandemic has further empowered the surveillance society and highlighted the complex issue of the close connection between control and healthcare. Building trust in medical experts, in the authorities, and in the surveillance technology itself that must be efficient and reliable, is of vital importance for gaining the public’s compliance with highly restrictive regulations, especially in democratic societies.
